# Genome of *Labrenzia* sp. PHM005 Reveals a Complete and Active *Trans*-AT PKS Gene Cluster for the Biosynthesis of Labrenzin

**DOI:** 10.3389/fmicb.2019.02561

**Published:** 2019-11-07

**Authors:** Dina Kačar, Carmen Schleissner, Librada M. Cañedo, Pilar Rodríguez, Fernando de la Calle, Beatriz Galán, José Luis García

**Affiliations:** ^1^Department of Microbial and Plant Biotechnology, Centro de Investigaciones Biológicas, Agencia Estatal Consejo Superior de Investigaciones Científicas, Madrid, Spain; ^2^Research and Development Department, PharmaMar S.A., Madrid, Spain

**Keywords:** pederin, biosynthesis, *trans*-AT PKS, expression, genetic modification

## Abstract

The complete genome of the strain *Labrenzia* sp. PHM005, a free-living producer of a pederin analog 18-*O*-demethyl pederin, hereinafter labrenzin, has been sequenced. This strain contains two replicons comprising a circular chromosome of 6,167,349 bp and a circular plasmid (named p1BIR) of 19,450 bp. A putative gene cluster responsible for the synthesis of labrenzin (*lab* cluster) has been identified showing that it encodes a *trans*-AT mixed type PKS/NRPS biosynthetic pathway that is responsible for the synthesis of pederin and possibly an onnamide analog. The putative boundaries of the *lab* gene cluster were determined by genetic comparisons with other related strains, suggesting that the cluster consists of a 79-kb region comprising 3 genes encoding multidomain hybrid polyketide synthase/non-ribosomal peptide synthetase (PKS/NRPS) proteins (PKS4, PKS/NRPS13, and PKS/NRPS15), and 16 auxiliary enzymes. Transcriptomic analyses suggest that all the genes of the cluster are expressed in our culture conditions (i.e., in minimal medium in the absence of any specific inducer) at detectable levels. We have developed genetic tools to facilitate the manipulation of this strain and the functional characterization of the cluster genes. We have created a site-directed mutant unable to produce pederin, demonstrating experimentally for the first time the role of the cluster in the synthesis of pederin. This work paves the way to unravel the clues of the biosynthesis of pederin family compounds and opens the door to modify and overproduce these anticancer drugs for industrial and pharmaceutical purposes.

## Introduction

Pederin is a natural polyketide extracted for the first time from insect genus *Paederus* Curt. (Coleoptera: Staphylinidae) as non-protein insect toxin (Frank and Kanamitsu, 1987). Twenty five million field-collected *Paederus fuscipes* were used to isolate pure pederin (Pavan and Bo, 1952) and its chemical formula was further determined (Cardani et al., 1965). It has been found that females of genera *Paederus* use pederin to chemically defend their offspring against predators (Kellner and Dettner, 1996). Pederin displays potent and selective bioactivities that have triggered biomedical interests (Soldati et al., 1966; Wan et al., 2011). It has a huge therapeutic potential because it is shown to be a highly potent anticancer agent (Richter et al., 1997). Closely related cytotoxic compounds like theopederins, mycalamides, and onnamides have been isolated from several genus of marine sponges (Pannell et al., 1988; Sakemi et al., 1988; Fusetani et al., 1992). It has been suggested that these molecules could be synthesized by symbiotic microbes existing in these marine invertebrates (Piel et al., 2004b).

The first insights into the true producers of pederin were obtained by cloning a fraction of the pederin biosynthesis genes (*ped* cluster) from total DNA of *P. fuscipes* beetles (Piel, 2002). The partial *ped* cluster that was initially located on a 54-kb region bordered by transposase pseudogenes encoded a mixed modular polyketide synthase/non-ribosomal peptide synthetase. The *ped* cluster was distributed among two distinct regions (*pedIJK* and *pedABCDEF*) of the genome from an uncultured bacterial symbiont of the insect that exhibited a similarity to genera *Pseudomonas* (Piel, 2002; Piel et al., 2004a, c). Supporting this finding, a dominant bacterium with the closest relationship to *P. aeruginosa* had previously been detected in *Paederus sabaeus* (Kellner, 2002).

An *onn* gene cluster closely related to *ped* genes was isolated later on from the metagenome of the marine sponge *Theonella swinhoei*, which was described as a source of onnamides and theopederins (Piel et al., 2004b). The isolated genes also belonged to a prokaryotic symbiont and the close similarity and complexity of the *ped* and *onn* biosynthetic systems suggested that they were derived from a common ancestral gene cluster.

Today, more than 30 members constitute the pederin-like family, containing almost an identical core region and variable polyketide or amino acid termini (Piel et al., 2005). Nonetheless, the gene clusters associated to their biosynthesis have mostly been identified in symbiont metagenomes, such as psymberin (Fisch et al., 2009), diaphorin (Nakabachi et al., 2013), and nosperin (Kampa et al., 2013) with an exception of cusperin (Kust et al., 2018) recently identified in a free-living cyanobacterium.

Moreover, the isolation of labrenzin from the culture of a marine heterotrophic α-Proteobacterium, *Labrenzia* sp. strain PHM005 isolated from marine sediment was reported (Schleissner et al., 2017; Cañedo et al., 2018). This was the first report of the production of a pederin-like compound by a free-living marine bacterium that could be cultured in the laboratory.

Despite the research carried out on the pederin biosynthetic pathways, direct evidence that such pathways were responsible for the pederin synthesis have not been published yet. Here, we describe the sequencing of the complete genome of *Labrenzia* sp. PHM005 and the identification of the *lab* gene cluster responsible for the synthesis of labrenzin. We have been able to manipulate genetically this strain to show experimentally a direct relationship of the *lab* cluster with the biosynthesis of labrenzin. The genomic and transcriptomic analyses carried out with this cultivable bacterium revealed new insights into the biosynthesis of pederin family compounds.

## Materials and Methods

### Culture Media and Bacterial Growth Conditions

Culture media used to grow *Labrenzia* sp. strain PHM005 (provided by PharmaMar) and to produce labrenzin were as follows: (i) Marine Broth (DIFCO 2216) (MB); (ii) Modified marine basal media (MBM) (Baumann and Baumann, 1981) containing NaCl (20 g/L) and 0.2% glucose as carbon source; (iii) MBM supplemented with vitamins (MBM + vit) [B12 (50 μg/L), panthotenic acid (50 μg/L), riboflavin (50 μg/L), pyridoxamine (10 μg/L), biotin (20 μg/L), folic acid (20 μg/L), nicotinic acid (50 μg/L), *p*-aminobenzoic acid (50 μg/L), thyamine (50 μg/L)]; (iv) MBM supplemented with biotin (20 μg/L) (MBM + BIOTIN); (v) MBM supplemented with thyamine (50 μg/L) (MBM + THYAMINE); (vi) MBM supplemented with biotin (20 μg/L) and thyamine (50 μg/L) (MBM + B + T).

The strain was usually grown overnight in 50 mL falcon tubes in MB at 30°C with shaking at 220 rpm. The overnight culture was washed in 0.85% NaCl solution and diluted to an optical density (OD_600_) ≈ 0.1 in 20 ml of fresh MBM medium. The OD_600_ was measured every 3 h during the cultivation.

### DNA Extraction, Sequencing and Genome Assembly

The strain was grown in MB for 24 h and the genomic DNA was extracted using Blood & Cell Culture DNA Mini Kit (Qiagen). The genome of a strain PHM005 was sequenced by a PacBio RSII sequencer and was assembled *de novo* in a single contig. Samples were prepared at PharmaMar and library construction, sequencing, data processing and assembly were performed by Macrogen.

Several sequencing errors were detected in the *trans*-AT PKS sequences that resulted in frameshifts that render several unexpected stop codons. The errors were corrected by Sanger sequencing of specific PCR amplicons surrounding the sequencing errors (Supplementary Figure S1). Primers used for PCR amplification for Sanger sequencing were designed using Primer3 implemented in the Geneious v.10.0.2 software (Supplementary Table S1). The circular map of a *Labrenzia* sp. PHM005 genome was generated using a CGView (Stothard and Wishart, 2005).

To determine the presence of plasmids in the strain PHM005 that cannot be detected by the PacBio sequencing method as well as to correct other putative PacBio sequencing errors, the genome of strain PHM005 was re-sequenced using an Illumina MiSeq system with a 300 nt pair-end library at the Complutense University of Madrid. The library was constructed following the manufacturer’s recommendations (Nextera DNA Flex, Illumina). The reads were trimmed and assembled into contigs using CLC Genomics Workbench software package (Quiagen).

The PacBio sequencing errors detected in the assembled chromosome were corrected manually after mapping the Illumina reads to PHM005 genome sequenced by PacBio using the Geneious v.10.0.2 software.

The Whole Genome projects have been deposited at GenBank under the accession CP041191 (*Labrenzia* sp. PHM005) and CP041190 (p1BIR). The DNA sequencing data have been deposited at sequence read archive (SRA) under the accession SRS5035208.

### Genomic Analyses

The genome of strain PHM005 was annotated using the NCBI Prokaryotic Genome Annotation Pipeline (PGAP). AntiSMASH v. 4.0. was used for a rough secondary metabolites cluster mining and the identification of the individual PKS/NRPS domains. The specificity of NRPS adenylation domains and *trans*-ATs was predicted based on a combination of tools implemented in antiSMASH 4.0 (Stachelhaus code, NRPS Predictor3, pHMM, SANDPUMA and Minowa).

Whole genome alignment of *Labrenzia* sp. PHM005, *L. alexandrii* DFL-11, *L. aggregata* RMAR6-6, *Labrenzia* sp. CP4 and *Labrenzia* sp. VG12 was performed using the progressive Mauve algorithm with default settings from the Geneious v.10.0.2. In addition, each strain was aligned separately to *Labrenzia* sp. PHM005 to calculate the minimum weight for Locally Collinear Blocks (LCB).

Average nucleotide identity (ANI) was calculated using reciprocal best hits (two-way ANI) between two genomic datasets in an online tool developed at Kostas lab (Rodriguez-R and Konstantinidis, 2016).

To establish gene cluster boundaries, the proximal DNA regions from each strain were aligned using MAFFT with the scoring matrix BLOSUM62 and the gap open penalty set to 1.

The dot plot of the protein alignment of PKSs and theirs Ped homologs was generated based on the score matrix BLOSUM62, window size 10 and threshold 23.

ECH1 protein domains were aligned using Geneious v.10.0.2 default parameters.

### Genetic Manipulations

The gene deletion was performed based on the I-*Sce*I endonuclease system using pSEVA312S and pSEVA428S plasmids (Martínez-García and de Lorenzo, 2011; Silva-Rocha et al., 2013). I-*Sce*I endonuclease causes double strand breaks that trigger DNA repair by homologous recombination. Primers PKS4 UP F and PKS4 UP R (5′-ACTA GTCTAGA CGCAGTCGTCCTGATGAGAT-3′ and 5′-ATCC CGAGCTCATGGGAACGTCCAGTATCGC-3′, respectively) were designed to amplify the upstream sequence (574 bp) flanking the deletion region. Primers PKS4 DOWN F and PKS4 DOWN R (5′-ATCCCGAGCTCGGTCGTATTC AACACCCGGT-3′ and 5′-TACCCAAGCTTAGGATCCTGC AAGAAGCCAC-3′, respectively; *Sac*I restriction site is underlined) were designed to amplify the downstream flanking sequence (637 bp). The upstream and downstream sequences were ligated flanking the *Sac*I restriction site and subcloned into the pCR^TM^-Blunt II-TOPO^TM^ plasmid (Invitrogen) to generate pTOPO-PKS4UP-DOWN plasmid. The pTOPO-PKS4UP-DOWN and suicide plasmid pSEVA312S were both digested with *Pst*I and *Spe*I. The UP-DOWN fragment released from the pTOPO-PKS4UP-DOWN was further ligated into previously digested pSEVA312S to generate pSEVA312SUP-DOWN and transformed into *E. coli* cc118λpir. Triparental conjugation with donor *E. coli* cc118λpir and helper *E. coli* HB101 harboring the plasmid pRK600 was used to transform *Labrenzia* sp. PHM005 for the genome integration of the suicide plasmid. The PHM005 transconjugants T1 were selected on the Petri plates with Marine Agar (MA) (Difco 2216) containing as selective antibiotics chloramphenicol (5 μg/mL) and kanamycin (10 μg/mL). Subsequently, pSEVA428S was transformed to PHM005 T1 by triparental conjugation and selected on the MA plates with selective antibiotics chloramphenicol (5 μg/mL), kanamycin (10 μg/mL) and streptomycin (50 μg/mL). The induction of I-*Sce*I endonuclease and the selection of the recombinant PHM005ΔPKS4 on MA, as well as the following curation of the pSEVA428S were performed as described (Martínez-García and de Lorenzo, 2011). The deletion recombinants were screened by PCR-amplification using external primers M1 F and M1R (5′-TCTTGGTGGACGAGACCAGT-3′ and 5′-GGCTTCGTGTGGTGAAATGC-3′, respectively) and the deletion (763 bp) was confirmed by Sanger sequencing.

### RNA Extraction and Gene Expression Analysis

Mutant strain PHM005ΔPKS4 was cultured in MBM + vit medium and the total RNA was extracted by High-pure Isolation Kit (Roche) after 24 h. Extracted RNA was additionally treated by DNA-*free* DNaseTreatment and Removal Reagents (Ambion). The RNA concentration was measured by using a NanoPhotometer (Implen). Reverse transcription of 1 μg of DNA-free RNA per sample was done using the Transcriptor First Strand cDNA Synthesis Kit (Roche) to generate cDNA and RT-PCR semi-quantitative analyses were carried out to check the mRNA levels. The *rpoD* gene was used as the housekeeping gene. Primers for RT–PCR for *Labrenzia* sp. PHM005 pederin cluster genes, as well as the housekeeping *rpoD* gene, were designed using Primer3 implemented by Geneious v.10.0.2 (Supplementary Table S2). Relative mRNA expression was visualized in 1.5% agarose gel. Negative controls contained RNA samples without the reverse transcriptase. The RNA extraction from the strain PHM005 for the transcriptomics analysis was performed in the early exponential phase (12 h) as described previously. Transcriptomics and bioinformatics analyses were carried out by Vertis Biotechnologie AG. The percentage of the mapped reads to annotated genes is 78.4, 80.8, and 77.0% for each replicate sample. The results represent a mean of three replicates.

### Extraction of Labrenzin and HPLC-MS Analyses

Strain PHM005 was cultured in media described above in three replicates and samples were extracted at 24 h, 72 h, and 6 days of growth. The mutant PHM005ΔPKS4 was cultured in MBM + vit. Samples were centrifuged and the supernatant was extracted twice with ethyl acetate (1:1, vol:vol) and the two organic fractions were combined and dried on a rotary evaporator. The dry matter was dissolved in methanol and filtered through 0.22-μm polytetrafluoroethylene syringe filters before HPLC-MS analysis. Labrenzin was purified and prepared as a standard by PharmaMar. HPLC-MS was carried out using a mass spectrometry system (Thermo Mod. Finnigan^TM^ LXQ^TM^) with on line HPLC Surveyor comprising pump with four separate solvent feeds (Surveyor MS pump Plus), an auto-sampler for multi sample analysis (Surveyor AS Plus), a Photo Diode Array detector (Surveyor PDA Plus) and a mass spectrometer LXQ equipped with linear ion trap. Ionization source used was electrospray ionization (ESI). Separation was performed on a C18 column (ZORBAX Eclipse plus C18, 5 μm, 4.6 × 250 mm, Agilent Technologies, Santa Clara, CA, United States). Solvent A was 100% water and solvent B was 100% acetonitrile. The flow rate was 1 ml min^–1^ and the gradient (per cent solvent A/B) was *t* = 2 min, 100% A; *t* = 5 min, 95% A; *t* = 25 min, 0% A; *t* = 27 min, 0% A; *t* = 30 min, 100% A; *t* = 35 min, 100% A.

## Results

### Genome Characteristics of *Labrenzia* sp. PHM005

**FIGURE 1:**
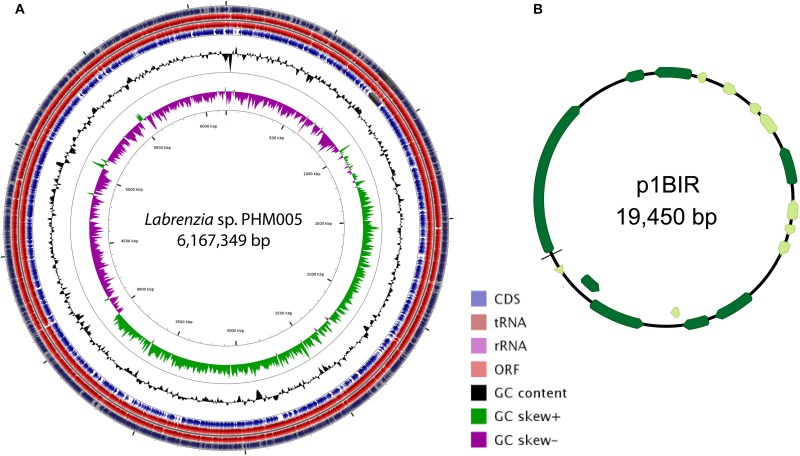
Circular map of the genome of *Labrenzia* sp. PHM005 and its plasmid p1BIR. **(A)** From outer to inner circles: The first four circles show the coding sequence (CDS), transfer ribonucleic acid (tRNA), ribosomal ribonucleic acid (rRNA), and open reading frame (ORF). The fifth circle represents the GC content (black). The sixth circle demonstrates the GC skew curve (positive GC skew, green; negative GC skew, violet). The genome position scaled in 500 kbp from base 1 is shown on the inner circle. **(B)** The circular map of plasmid p1BIR (Geneious version 10.0 created by Biomatters. Available from http://www.geneious.com). Dark green arrows represent genes coding for proteins with homology in other species from NCBI database and light green represents hypothetical proteins.

The complete genome sequence of *Labrenzia* sp. strain PHM005 revealed two replicons comprising a circular chromosome of 6,167,349 bp (accession CP041191) and a circular plasmid (named p1BIR) of 19,450 bp (accession CP041190) shown in Figure 1. The genome average G + C content is 55%, contains 51 tRNAs and 3 copies of rRNA sequences (5S-23S-16S) (Figure 1). The genome contains 5,988 coding sequences that were classified by Rapid Annotation Subsystem Technology (RAST) Server (Aziz et al., 2008) into 483 subsystems and 26 categories (Supplementary Figure S2). Functional comparison of genome sequences in the RAST database revealed *Labrenzia alexandrii* (strain DFL-11) (score 548) as the closest neighbor of strain PHM005, followed by *Labrenzia aggregata* (strain IAM 12614) (score 539), *Roseibium* sp. (strain TrichSKD4) (score 328), *Agrobacterium tumefaciens* (strain C58) (score 207) and *Sinorhizobium meliloti* (strain 5A14) (score 198).

The genomes of five *Labrenzia* species, i.e., *Labrenzia* sp. (strain PHM005) (accession CP041191), *L. alexandrii* (strain DFL-11) (accession CM011002), *L. aggregata* (strain RMAR6-6) (accession CP019630), *Labrenzia* sp. (strain CP4) (accession CP011927) and *Labrenzia* sp. (strain VG12) (accession CP022529), were analyzed for average nucleotide identity (ANI) to provide intraspecies and interspecies relationships (Supplementary Figure S3). Strain PHM005 appears to be most similar to *L*. *alexandrii* (strain DFL-11) although both genomes do not seem to belong to the same species since the ANI value is below 95% (Goris et al., 2007). Nonetheless, only *L. aggregata* (strain RMAR6-6) and *Labrenzia* sp. (strain CP4) most likely belong to the same species showing an ANI value of 97.76%.

Plasmid p1BIR (accession CP041190) has a G + C content of 48.5% and contains 17 ORFs most of them coding for putative replication and conjugation related proteins (Figure 1 and Supplementary Table S3). The plasmid carries two putative Abi family proteins described to be involved in bacteriophage abortive infection resistance systems (Garvey et al., 1995). A plasmid copy number of 59 was calculated taking into account the genome and plasmid size and the number of Illumina reads covering them, *i. e*., 3,820,045 reads and 712,018 reads, respectively (SRA accession SRS5035208).

### Analysis of the Secondary Metabolite Gene Clusters in *Labrenzia* sp. PHM005

The circular genome of *Labrenzia* sp. PHM005 was set for a secondary metabolite gene cluster mining using an online platform antiSMASH 4.0. In total, 101 gene clusters have been identified: (i) one bacteriocin or other unspecified ribosomally synthesized and post-translationally modified peptide product (RiPP) cluster; (ii) 4 putative fatty acid clusters; (iii) 4 putative saccharide clusters; (iv) 89 putative clusters of unknown type; (v) one putative polyhydroxyalkanoate biosynthetic gene cluster; (vi) one putative mixed PKS/saccharide cluster; (vii) one *trans*-AT PKS/NRPS cluster. The similarity of these clusters with the known clusters from MIBiG repository is shown in Supplementary Table S4. Additionally, the rest of the clusters were screened for similarity in other microorganisms and we have counted 17 clusters which lie within the order of *Rhizobiales* sharing from 10 to 32% of cluster genes (Supplementary Table S5).

### Proposed Modular Biosynthesis of Labrenzin

**FIGURE 2:**
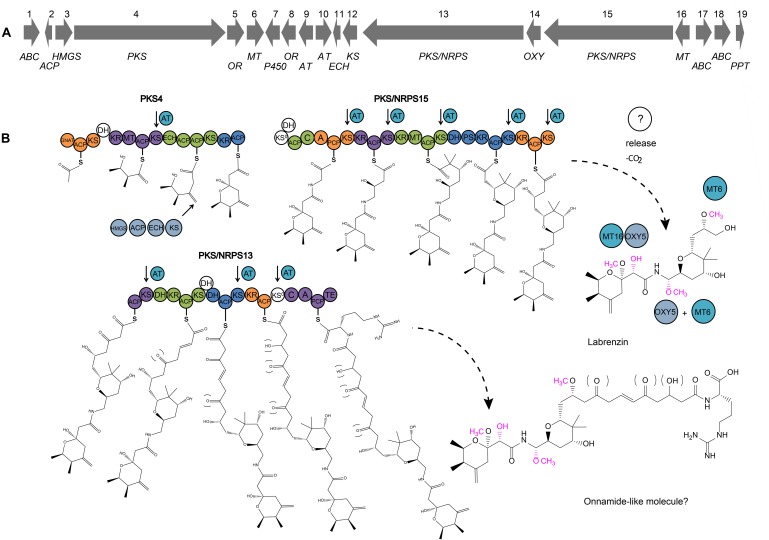
**(A)** Map of the *lab* cluster comprising genes for labrenzin production. **(B)** Scheme predicting the pederin/onnamide modular biosynthesis in a free-living Alfaproteobacterium *Labrenzia* sp. PHM005. Specific domains have been identified and analyzed using antiSMASH. Domains (shown in circles) that are part of the same module are represented in the same color, less the putative non-functional domains in white. Module boundaries are defined as described by Vander Wood and Keatinge-Clay (2018). GNAT, GCN5-related *N*-acetyltransferase domain; ACP, acyl carrier protein domain; KS, ketosynthase domain; KS^0^, non-elongating ketosynthase domain; KR, ketoreductase domain; MT, methyltransferase domain; ECH, enoyl- CoA hydratase/isomerase domain; A, adenylation domain; AT, *trans*-acyltransferase; C, condensation domain; DH, dehydratase domain; PS, pyran synthase; PCP, peptidyl carrier protein domain; TE, thioesterase domain; OR, oxidoreductase; MT, methyltransferase. Putative trans-AT docking site associated with its respective KS is indicated by arrows. Two pathways are indicated: the synthesis of the labrenzin and the onnamide A questioned to be the complete molecule of the proposed pathway. Putative functional groups are marked inside brackets and putative modifications by tailoring enzymes are colored in pink.

The *trans*-AT PKS/NRPS cluster, further named *lab* cluster, consists of a 79-kb region (776,792–855,905 bp) comprising 3 genes encoding multi-domain PKS/NRPS proteins (PKS4, PKS/NRPS13, and PKS/NRPS15), and 16 auxiliary enzymes (Figure 2A).

The core pederin molecule is constructed following the “assembly line” biosynthesis by modular PKSs/NRPSs and further modified by tailoring enzymes as proposed in the scheme (Figure 2B). The structure of biosynthesis intermediates of labrenzin has been deducted from the specificity of enzymatic domains of each PKS/NRPS module. The domain analysis based on antiSMASH results and the described conserved motifs (El-Sayed et al., 2003) of the three PKS/NRPS are listed in Supplementary Table S6. According to the modular structure predicted by antiSMASH v.4.0., the PKS4 from strain PHM005 consists of a “starter” module 0 with a GCN5-related *N*-acetyltransferase family (GNAT) domain that catalyzes the decarboxylation of malonyl-CoA to generate acetyl-CoA and its translocation to the ACP unit. After that, three elongating β-ketoacyl synthases (KSs) incorporate the 3 malonyl-CoA building units. Compared to PedI from *P. fuscipes* symbiont (Piel et al., 2004a), the first module of PKS4 contains an additional dehydratase (DH) domain, most probably inactive, since it lacks the typical conserved motif HXXXGXXXXP (El-Sayed et al., 2003). Also, the presence of only one ECH domain in the third module does not appear to affect the structure of the first pyran ring of the pederin core structure.

The domain architecture of the PKS/NRPS15 from strain PHM005 is almost identical to PedF (Piel, 2002). The singularity of PKS/NRPS15 is the presence of a putative inactive DH domain attached to the first non-elongating KS^0^, while in PedF there is one putative inactive DH domain in the last module.

According to antiSMASH predictions, the PKS/NRPS13 from strain PHM005, follows the domain order described in PedH, which is hypothesized by Piel (2002) to synthesize the chain containing the terminal arginine residue found in the onnamide analogs (Matsunaga et al., 1992). However, there are a few exceptions. Substrate predictions of PKS/NRPS13 by pHHM and SANDPUMA indicate arginine and alanine, respectively, as candidate amino acids for the chain terminal of the putative onnamide-like molecule, therefore we cannot conclude with accuracy the last amino acid incorporation. PKS/NRPS13 also possesses another, probably inactive DH domain in the third module. The last non-elongating KS lacks the histidine residue and the ACP located downstream lacks the conserved GxDS motif (El-Sayed et al., 2003).

### Pederin Cluster Boundaries

**FIGURE 3:**
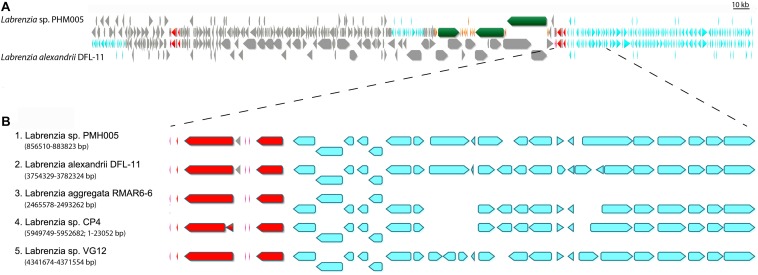
Representation of the *lab* gene cluster and its bordering genes distributed among the selected *Labrenzia* species genomes. **(A)** MAFFT alignment of *Labrenzia alexandrii* DFL-11 and *Labrenzia* sp. PHM005. Coding sequences are represented by arrows: gray, CDS; red, ribosomal DNA; green and orange, *lab* gene cluster; light blue; identical genes. **(B)** MAFFT alignment and localization of downstream bordering genes in 5 *Labrenzia* species.

The genome of *L. alexandrii* (strain DFL-11) was used to estimate the size and the putative boundaries of the *lab* cluster in the genome of strain PHM005. The alignment of both genomes is shown in Figure 3. This alignment revealed that the *lab* cluster has been probably inserted within two rDNA regions. The putative cluster limits were further compared with the genomes of 3 other species of *Labrenzia*, i.e., *L. aggregata* (strain RMAR6-6) (accession CP019630), *Labrenzia* sp. (strain CP4) (accession CP011927) and *Labrenzia* sp. (strain VG12) (accession CP022529). Using the five previously described *Labrenzia* species we performed a whole genome alignment using a progressive Mauve algorithm from the Geneious version 10.0.2 (Supplementary Figure S4). According to these algorithms, *L. alexandrii* (strain DFL-11) showed the highest local collinearity number (Supplementary Table S7). Interestingly, the genes located downstream from the *lab* gene cluster were found in the same orientation in all 5 species. The similarity and the corresponding gene positions in each species are shown in Figure 3B. On the contrary, the genes located upstream the *lab* cluster were only found in *L. alexandrii* (strain DFL-11) (gene position at genome between 3,628,890 bp and 3,648,023 bp). Accordingly, to these comparisons, the homologous genes present in at least two genomes were discarded as candidates for the biosynthesis of labrenzin allowing us to fix the putative boundaries.

### Comparative Analysis With *ped, onn* and *dip* Gene Clusters

**FIGURE 4:**
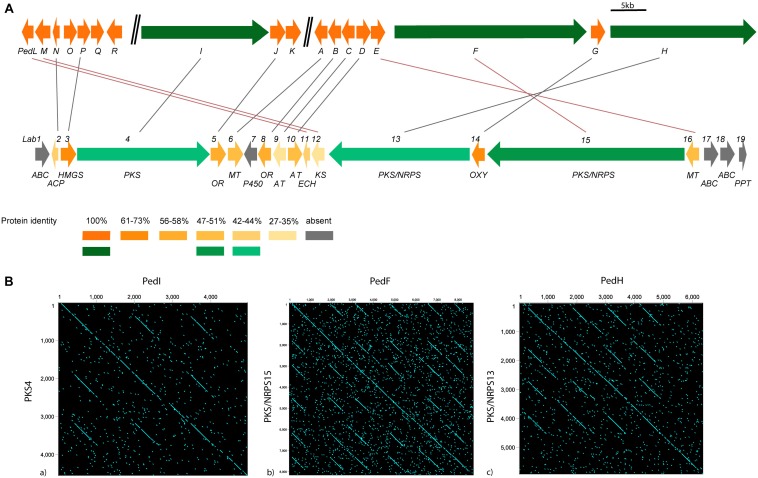
Comparative analysis of a pederin cluster from a symbiont bacterium of *P. fuscipes* and the free-living Alphaproteobacterium *Labrenzia* sp. PHM005. Modified scheme of the pederin cluster as described from a: **(A)** bacterium symbiont of *P. fuscipes* (Piel et al., 2004b) (above) and the newly described labrenzin cluster from *Labrenzia* sp. PHM005 (below). Homologous genes between two clusters are indicated by lines. Protein identity is visualized using colored scale that matches the identity percentage of all the genes from the cluster, using as reference the symbiont bacterium of *P. fuscipes* genes. PKS and NRPS genes are shown in green and tailoring enzymes in orange and gray. ABC, transporter; ACP, acyl carrier protein; AT, acyltransferase; ECH, enoyl- CoA hydratase/isomerase domain; HMGS, hydroxymethylglutaryl-CoA synthase; KS, ketosynthase; MT, methyltransferase; OR, oxidoreductase; OXY, oxygenase; P450, cytochrome hydroxylase; PPT, phosphopantetheinyl transferase. **(B)** Graphical representation of the homology using dotplotting between PKS 4, 13, and 15 and PedI, PedH, and PedF, respectively.

The results of the BLAST protein homology analysis indicate the similarity with pederin, onnamide and diaphorin gene clusters (Supplementary Table S8), i.e., other members of the pederin-family biosynthetic clusters. Using as a reference the genes of the pederin cluster of the symbiont from *P. fuscipes*, the homologous genes and their protein identities of the corresponding genes of *Labrenzia* sp. PHM005 are visualized in Figures 4A,B. The first observed difference between the two almost identical biosynthetic systems is the gene distribution in their host genomes. In the symbiont bacterium of *P. fuscipes*, the pederin genes are clustered in three genomic islands flanked by IS elements and transposons (Piel et al., 2004c, 2005). However, the pederin biosynthetic genes of strain PHM005 are clustered together, suggesting its horizontal gene transfer, a very common phenomenon in bacteria as it is hypothesized by Roth and Lawrence (1996).

The *lab* gene cluster of strain PHM005 comprises 3 putative ABC transporters, one cytochrome P450 and one 4′-phosphopantetheinyl transferase, which have not been identified in *P. fuscipes* or *T. swinhoei*. On the other hand, there are three proteins in the *P. fuscipes* symbiont, that are absent in *Labrenzia* sp. PHM005, i.e., a putative PedQ esterase, a PedR regulator and a protein of unknown function named PedK in *P. fuscipes* or OnnE and OnnF in *T. swinhoei* (Piel et al., 2004b). The tailoring enzymes in strain PHM005 showed protein identity < 60%, with the exception of the hydroxymethylglutaryl-CoA synthase (HMGS) and the oxygenase (OXY) which have 61 and 73% identity, respectively. It has been demonstrated that β-branching enzymes known as HMGS cassettes introduce alkyl side-chains into β-position and they usually comprise HMGS, ACP, ECH and stand-alone KS modules (Buchholz et al., 2010). Among the tailoring enzymes in *lab* cluster, there is a presence of HMGS, stand-alone ACP, KS and ECH, two putative acetyl-transferases (AT) and one oxidoreductase (OR).

**FIGURE 5:**
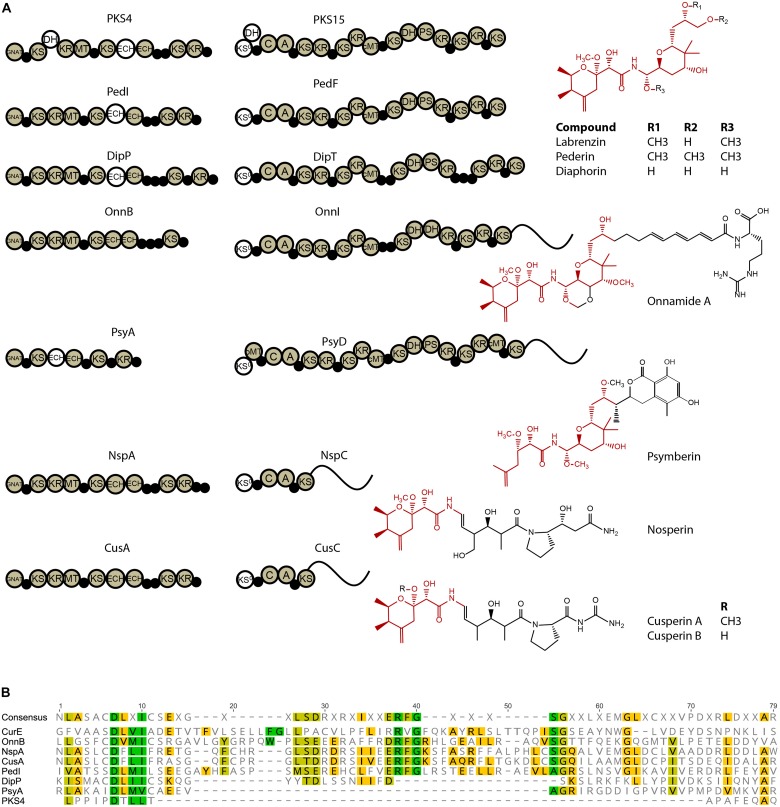
**(A)** Modular scheme and domain comparison of PKS/NRPSs responsible for the biosynthesis of pederin family compounds isolated from organisms of different ecological niches: labrenzin, *Labrenzia* sp. PHM005; pederin, *P. fuscipes* symbiont; diaphorin, *Profftella armatura*; onnamide A, *T. swinhoei* symbiont; psymberin, *Psammocinia* sp. symbiont; nosperin, Nostoc sp.; cusperin, *Cuspidothrix issatschenkoi* (top to bottom). Common core structure of the respective polyketides is colored red. **(B)** Alignment (aa) of putative ECH1 domains from the first PKS in onnamide A, nosperin, cusperin, pederin, diaphorin, psymberin and labrenzin (top to bottom).

Another difference regarding the *ped* cluster is the number of methyl-transferases (MT). *Ped* cluster described in the beetle symbiont comprises 3 MTs (PedA, PedE and PedO) while *lab* cluster in PHM005 strain has only two (MT6 and MT16). In comparison with pederin (4 *O*-methyl groups), labrenzin has only 3 *O*-methyl groups (see Figure 5A). Interestingly, labrenzin lacks the methylation at the terminal C18-OH group. Previous *in vitro* experimental analysis established that PedO is responsible for the methylation of the C18-OH group in pederin and it was hypothesized that PedA or PedE could have a dual function (Zimmermann et al., 2009). According to BLAST identity (Supplementary Table S8), MT16 shows 51% identity to PedE, while MT6 shows 47% and 43% identity to PedA and PedO, respectively. OnnG and OnnH from the onnamide cluster are homologous to PedA and PedE, respectively, but there is no Onn homolog for PedO (Piel et al., 2004b). Another close pederin analog, diaphorin, has only one *O*-methyl group because diaphorin gene cluster lacks the PedA and PedO orthologs (Nakabachi et al., 2013). Nonetheless, the BLAST identity between DipM and PedE is 60%.

According to these comparisons and BLAST identities with PedA, PedE, and PedO, we suggest that MT16 and MT6 are functional analogs of PedE and PedA, respectively. In this case, MT16 might have a dual function as PedE and the lack of PedO analog results in the absence of methylation at the terminal C18-OH group.

The three PKS/NRPS genes show less than 50% identity, but conserving the modular repeats of the enzymes as visualized by dot-plot (Figure 4B).

### PKS/NRPS Domain Comparison Between the Known Pederin Family Compounds

Apart from pederin and onnamide gene clusters mentioned above, other biosynthetic gene clusters of compounds from the pederin family, such as those described for the biosynthesis of diaphorin, psymberin, nosperin and cusperin were inspected for the PKS/NRPS domain architecture using the antiSMASH v.4.0 software (Figure 5A). The starting PKS responsible for the synthesis of the first pyran ring of pederin is conserved in all these compounds, except in the psymberin starting PKS which lacks the second module resulting in the absence of three C atoms. There is also a difference in the number of the tandem ACP domains found in the third module of the starting PKS.

In addition, the third module comprises two enoyl-CoA hydratase (ECH1 and ECH2) domains in OnnB, while the rest of the starting PKSs contain a single ECH domain based on antiSMASH analysis. The ECH domains can exist as separate enzymes, as a part of the HMGS-cassette, or as embedded domains in the PKSs, like ECH2 in curacin A (Liu et al., 2006). It is hypothesized that the ECH domains are responsible for the exomethylene group formation in the pederin pyran structure (Piel et al., 2004c). The amino acid sequence of the putative ECH1 domains in all 7 PKSs were aligned using as reference the characterized ECH1 functional domain (CurE) from the curacin A biosynthetic pathway (Liu et al., 2006) (Figure 5B). The OnnB, NspA, CusA, and PedI maintain the conserved amino acid motifs, while DipP, PsyA, and PKS4 have deleted the regions containing the conserved motifs of ECH1, suggesting that they might not be functional and that only one functional ECH domain is enough to generate the corresponding compounds.

### Lab Cluster Expression Analysis, Functional Gene Analysis and Labrenzin Production

**FIGURE 6:**
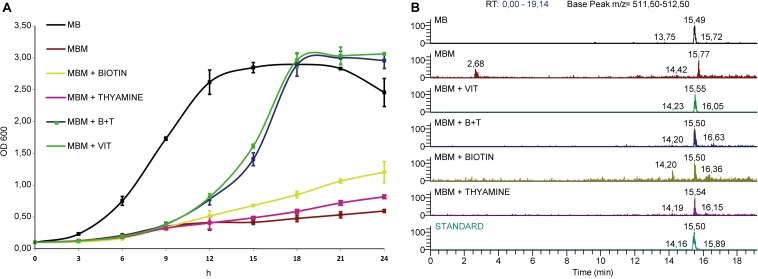
Growth curves and extracted ion chromatograms of culture extracts. **(A)** Strain is grown in Marine Broth (MB) and Marine Basal Medium (MBM) supplemented with different vitamin mixtures as indicated in the legend. **(B)** MS chromatograms showing the labrenzin extracted from different media after 24 h of cultivation (from up to down): MB; MBM; MBM + VIT; MBM + B + T; MBM + Biotin; MBM + Thyamine; standard.

We have determined that *Labrenzia* sp. PHM005 can grow in the rich medium marine broth (MB) and the defined medium Marine Basal medium (MBM) containing glucose as carbon source and supplemented with vitamins as described in Experimental procedures (Figure 6). Labrenzin was detected by mass spectrometry in both media within the first 24 h of growth (Figure 6).

**FIGURE 7:**
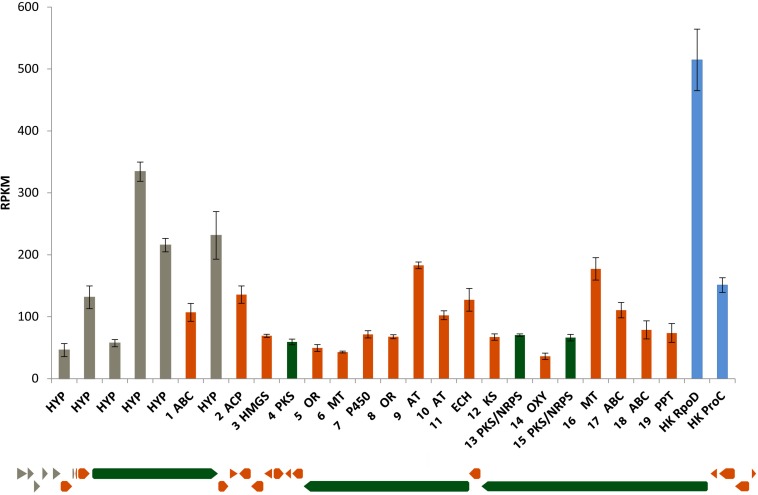
*Lab* cluster gene expression levels in early exponential phase cultured in MBM + vit medium. The order of the bars follows the order of the genes (from **left** to **right**). The results represent the mean of three replicates. Blue bars represent housekeeping genes RpoD and ProC.

The MBM + vit medium was chosen for growing the bacterium to carry out the transcriptomics analysis. Figure 7 shows the expression levels of *lab* cluster genes and the housekeeping genes in the early exponential phase. All the *lab* cluster genes are expressed suggesting that these genes are activated during the exponential phase of growth and none of the *lab* cluster genes is completely silenced.

To investigate the association between the described gene cluster and the production of labrenzin we performed a gene knockout by homologous recombination where the sequence comprising the putative promoter region of the gene encoding PKS4 and the region encoding the GNAT domain of the first module of PKS4 was deleted to generate a non-producing mutant (ΔPKS4) (see Experimental procedures section).

**FIGURE 8:**
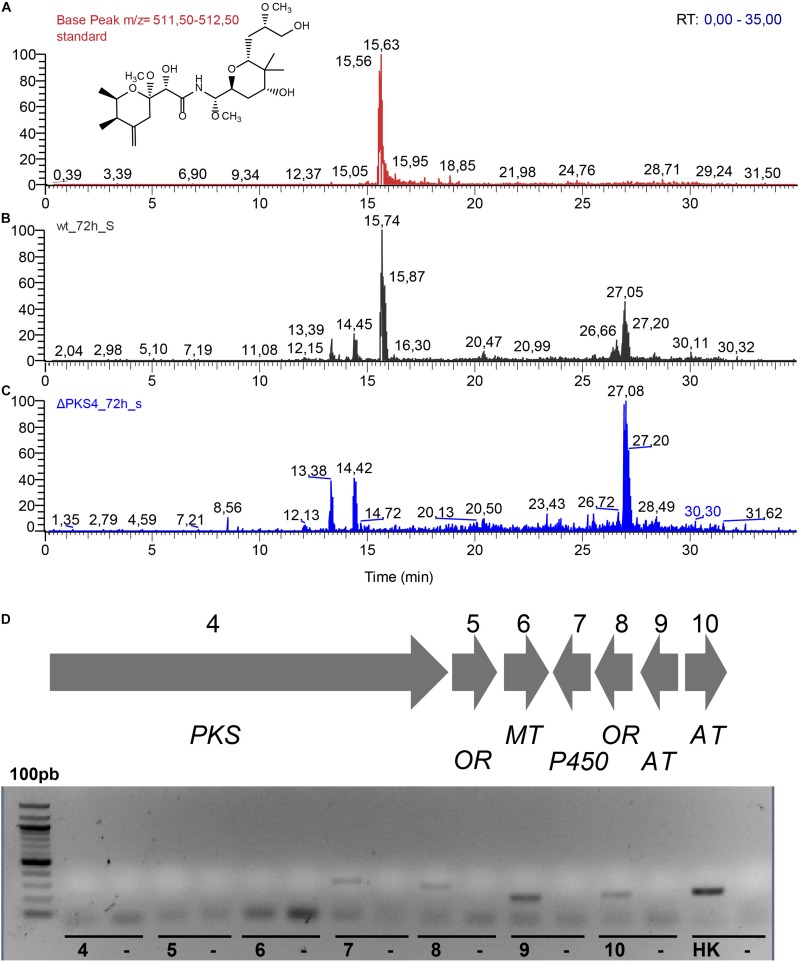
**(A)** Extracted ion chromatograms of the supernatant extracts obtained after 72 h of cultivation of *Labrenzia* sp. PHM005 in MBM + vit medium: **(A)** labrenzin standard; **(B)** wild type; **(C)** ΔPKS4. **(D)** RT-PCR analysis of the mRNA expression of *lab* gene cluster in the PHM005ΔPKS4 mutant cultured in MBM + vit medium. The numbers represent genes from the cluster. Each control lane contains a sample lacking a reverse transcriptase.

The analyses of the culture extracts of the wild type and mutant strain are shown in Figures 8A–C. The MS ionization pattern of labrenzin, as purified standard or present in the culture extracts, is shown in the Supplementary Figure S5. As expected, the mutant strain was unable to produce labrenzin. The gene expression analysis performed with the mutant strain suggested that the deletion has produced a polar effect on the expression of the genes coding for putative oxidoreductase (5) and methyl-transferase (6) that are located downstream of the gene encoding PKS4, suggesting that the three genes are transcribed as an operon (Figure 8D).

## Discussion

The potent and selective bioactivities of pederin family compounds have triggered their pharmaceutical interest during the last years mainly as drug candidates for cancer therapy (Wan et al., 2011). The production of pederin analogs had been ascribed to non-cultivable endosymbiont bacteria until the recent discovery of a pederin analogs produced by the free-living bacterium *Labrenzia* sp. PHM005 (Schleissner et al., 2017). These compounds were only obtained after extraction from insects or marine organisms (Helfrich and Piel, 2016), and by chemical synthesis through a multi-step process due to its complex structure (Wu et al., 2011). The pederin and onnamide biosynthetic clusters described so far have been reconstructed using partial sequences obtained from metagenomics analyses and it had not been possible to fix precisely the boundaries of these clusters as well as to carry out regulatory and expression analyses. The discovery of the labrenzin produced by the cultivable strain *Labrenzia* sp. PHM005 has opened a new technical window not only to understand its biosynthesis and regulation, but also to apply biotechnological tools for industrial purposes and for developing large-scale production of these anti-cancer agents. In this sense, we have developed genetic tools to carry out metabolic engineering including the possibility of using for the first time in these genera replicative plasmids such as pSEVA428S and others pSEVA plasmids with different selection markers and replicative origins (data not shown). Therefore, this work has laid the foundations and expanded the tools to explore this organism.

Based on *in silico* genomic approaches we have identified and described a *trans*-AT PKS/NRPS cluster that is responsible for the synthesis of labrenzin a pederin analog in *Labrenzia* sp. PHM005. This *lab* cluster consists of three main PKSs/NPRSs genes complemented by several tailoring genes that might be responsible not only for the synthesis of labrenzin but also for the synthesis of an onnamide analog. Interestingly, the partially published sequence of the homologous onnamide gene cluster (*onn* cluster) found in the metagenome of the sponge *T. swinhoei* contains all the PKS genes in a single region (Piel et al., 2004b) like in the strain PHM005. It has been postulated that the diverse onnamide and pederin analogs produced by the sponge might be generated by the same *onn* cluster by using alternative synthetic mechanisms (Piel et al., 2004b). Since the *lab* cluster of strain PHM005 also contains the PKS/NRPS protein assigned to the biosynthesis of onnamide (Piel, 2002), it can be classified as a pederin and onnamide gene cluster. However, we have not detected onnamide analogs under our cultivation conditions so far.

Our data suggest that all the genes encoding the *trans*-AT PKS/NRPS of the cluster are expressed during the growth of *Labrenzia* sp. PHM005 and thus, it could be assumed that an onnamide analog should be synthesized. However, we cannot discard the possibility of an oxidative cleavage by OXY, a putative monooxygenase homologous to PedG, releasing the pederin intermediate as discussed previously (Piel, 2010). The putative monooxygenase is hypothesized to either cleave off the intermediate stalled at the terminus module of PedF, homologous to PKS15 in *lab* cluster, or process the already synthetized onnamide-like precursor. Furthermore, recent *in vitro* studies on SNAC and ACP derivatives demonstrated that PedC has proofreading activity in PKS systems and is able to hydrolase thioesters of different chain lengths (Dimitrova et al., 2017). The existence of the second AT in the *lab* cluster, homologous to PedC, newly denominated as acyl hydrolase (AH) by Dimitrova and co-workers could be another possible candidate for an intermediate release.

The *lab* cluster of *Labrenzia* sp. PHM005 contains only two MTs in contrast with the three MTs present in the cluster described by Piel (2002). This observation is consistent with the fact that labrenzin lacks the methylation at the terminal C18-OH and reinforces the hypothesis of Zimmermann et al. (2009), which suggested that PedO was responsible for the methylation of C18-OH and at least one of the two MTs, PedA or PedE, should have a dual function to be able to methylate two different OH groups. The functionality of tailoring enzymes of *lab* cluster is currently under study in our laboratory.

The biosynthesis of secondary metabolites is influenced by a wide variety of environmental and physiological signals, presumably reflecting the range of conditions that trigger their production in nature. Our data show that all genes from *lab* cluster are transcribed in our laboratory conditions at a detectable level with the sole exception of the gene encoding the HMGS enzyme. This apparent constitutive expression of the cluster could be explained with the absence of any specific regulator within the cluster or even closed to the cluster, in contrast with the finding in the *ped* cluster described in the *P. fuscipes* symbiont that codes for a putative regulator PedR belonging to the LysR family (Piel et al., 2004b).

The genetic manipulation of *Labrenzia* sp. PHM005 strain has been our first milestone toward the unraveling the biosynthesis of pederin and pederin-like compounds. We constructed a mutant strain deleting the promoter region and a GNAT module of the PKS4. The resulting strain was unable to produce pederin demonstrating for the first time that the *lab* cluster was certainly involved in the pederin biosynthesis. The genetic manipulation of *Labrenzia* sp. PHM005 opens the possibility of studying the pathway at a molecular level, not only to unravel the biosynthetic mechanisms for pederin synthesis but also to generate new pederin family analogs for pharmaceutical purposes.

There are remarkable challenges ahead in understanding the regulatory cascades that link environmental and developmental signals to pleiotropic and ultimately pathway-specific regulatory genes for secondary metabolism. The availability of the entire genome sequence of *Labrenzia* sp. PHM005 and the possibility to develop transcriptome analyses will definitively provide some clues to fully exploit the industrial potential of this strain.

## Conclusion

The identification of the *lab* gene cluster responsible for the synthesis of labrenzin in the cultivable bacterium *Labrenzia* sp. PHM005 together with the development of the metabolic engineering tools described in this work will facilitate not only the understanding of the mechanisms involved in the biosynthesis of pederin and onnamide analogs that are extremely intriguing, but also the production by fermentation at industrial scale of these compounds that can be very useful to develop a new family of drugs based on one of the most active anticancer molecules described so far.

## Data Availability Statement

The Whole Genome projects have been deposited at GenBank under the accession CP041191 (*Labrenzia* sp. PHM005) and CP041190 (p1BIR). The DNA sequencing data have been deposited at sequence read archive (SRA) under the accession SRS5035208.

## Author Contributions

FC and JG conceived and designed the study. DK performed the experiments and analyzed the data. BG and CS participated in the experiment design. LC developed the HPLC-MS method and prepared the standard. PR extracted the DNA for PacBio sequencing. DK and JG wrote the manuscript. BG and CS contributed to preparing the final version of the manuscript. All authors have read and approved the final manuscript.

## Conflict of Interest

FC, LC, and PR were employed by the company PharmaMar S.A. The remaining authors declare that the research was conducted in the absence of any commercial or financial relationships that could be construed as a potential conflict of interest.

## Supplementary Material

FIGURE S1 Correction of a parted pederin family gene cluster sequence by Sanger sequencing.

FIGURE S2 The genome of *Labrenzia* sp. PHM005 annotated using the RAST. The green part in the bar chart at the leftmost position corresponds to the percentage of genes assigned to different subsystems. The pie chart demonstrates the percentage of each subsystem in *Labrenzia* sp. PHM005. The gene counts of the each subsystem is shown on the right.

FIGURE S3 Average nucleotide identity (ANI) comparison shows interspecies and intraspecies relationships of five strains in the *Labrenzia* genera. Identity scale: Green = 100%; orange > 95%; yellow ≥ 80%; gray < 80%.

FIGURE S4 Whole genome alignment using by progressive Mauve algorithm (Genious version 10.0.2), from up to the bottom: *L. aggregata* RMAR-6, *Labrenzia* sp. CP4, *Labrenzia* sp. VG12, *Labrenzia* sp. PHM005, *L. alexandrii* DFL-11.

FIGURE S5 Specific MS ionization pattern of labrenzin in culture extracts of the wild type PHM005 and the PHM005ΔPKS4 mutant after 72 h of cultivation in MBM + vit medium. **(A)** Pure labrenzin (standard) fragmentation patern at RT = 15,72 min. **(B)** Labrenzin fragmentation patern at RT = 15,72 min extracted from the wild type strain culture. **(C)** Background fragmentation patern at RT = 15,72 min extracted from the PHM005ΔPKS4 mutant strain culture.

TABLE S1 Primers used for the cluster sequence correction.

TABLE S2 Primers used in qPCR.

TABLE S3 Coding sequences of plasmid p1BIR.

TABLE S4 List of clusters homologous to those identified in *Labrenzia* sp. PHM005 by antiSMASH 4.0.

TABLE S5 Homologous gene clusters in other microorganisms according to ClusterBlast by antiSMASH 4.0.

TABLE S6 Domain specificity of the three PKS and mixed PKS/NRPS.

TABLE S7 Four *Labrenzia* genomes aligned to *Labrenzia* sp. PHM005 using progressive Mauve algorithm individually.

TABLE S8 BLAST homology of the *Labrenzia* sp. PHM005 *trans*- AT PKS gene cluster.
