# Supplementary figures and images for: Genome of Labrenzia sp. PHM005 Reveals a Complete and Active Trans-AT PKS Gene Cluster for the Biosynthesis of Labrenzin
Source: Front Microbiol. 2019 Nov 7;10:2561. doi: 10.3389/fmicb.2019.02561 (PMC6855096; doi:10.3389/fmicb.2019.02561)

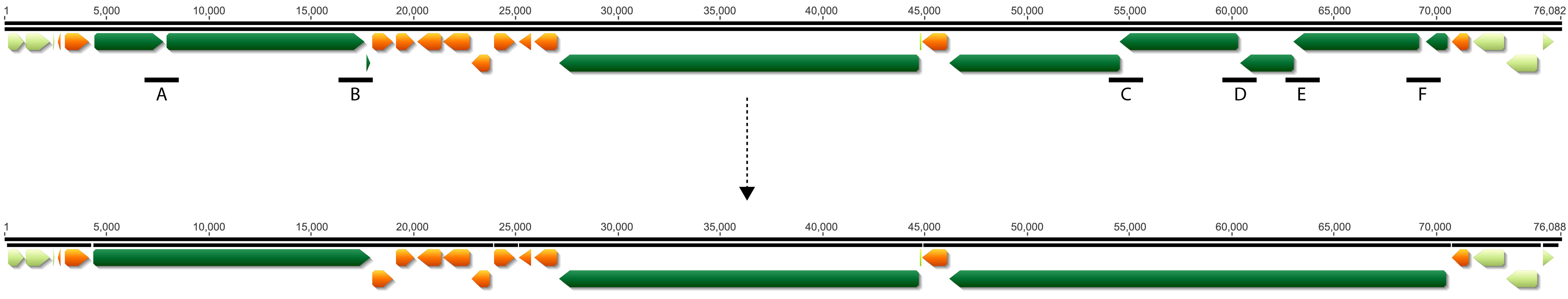

Supplement: FIGURE S1 — Correction of a parted pederin family gene cluster sequence by Sanger sequencing. [file Image_1.JPEG]

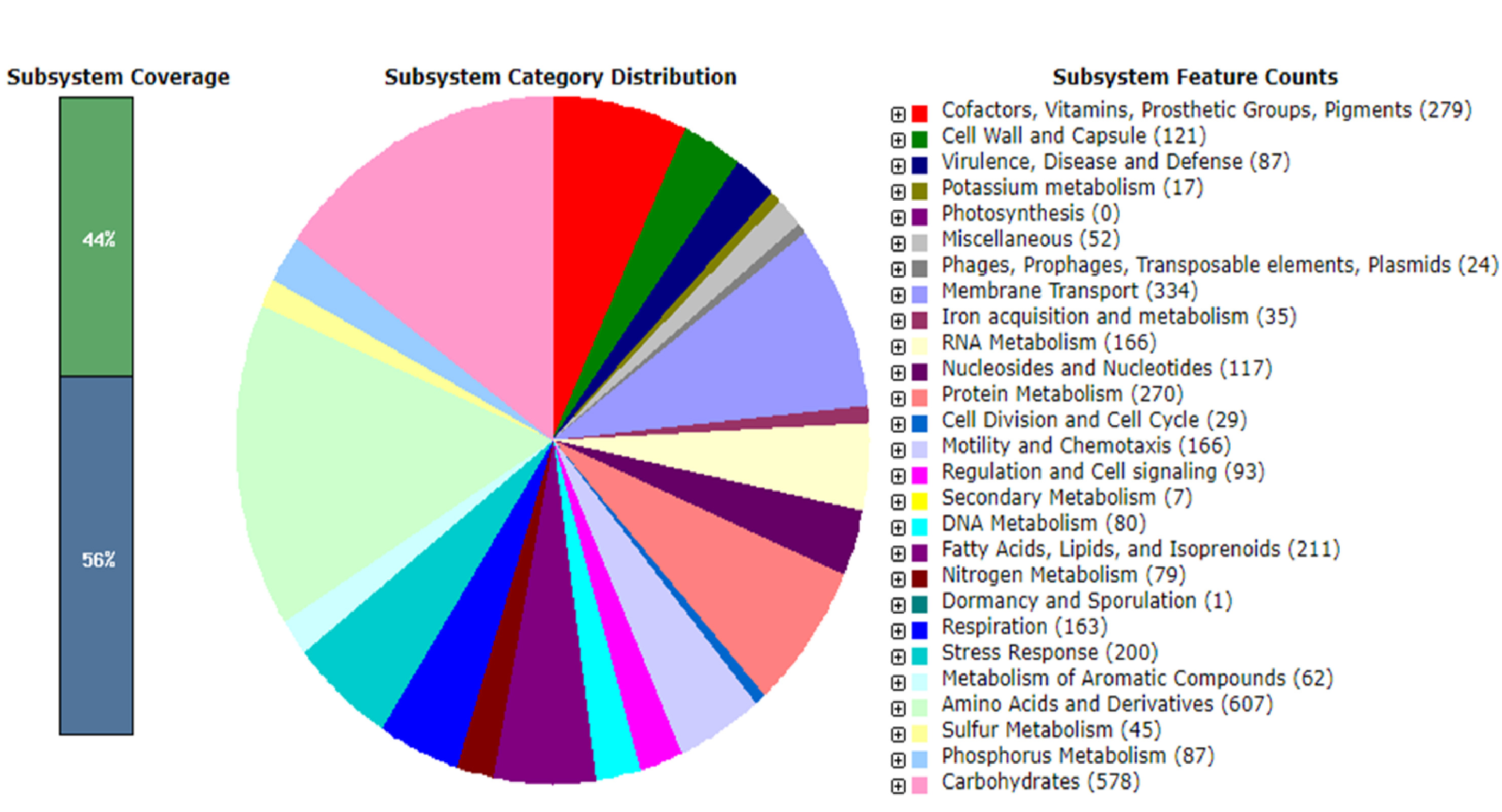

Supplement: FIGURE S2 — The genome of Labrenzia sp. PHM005 annotated using the RAST. The green part in the bar chart at the leftmost position corresponds to the percentage of genes assigned to different subsystems. The pie chart demonstrates the percentage of each subsystem in Labrenzia sp. PHM005. The gene counts of the each subsystem is shown on the right. [file Image_2.JPEG]

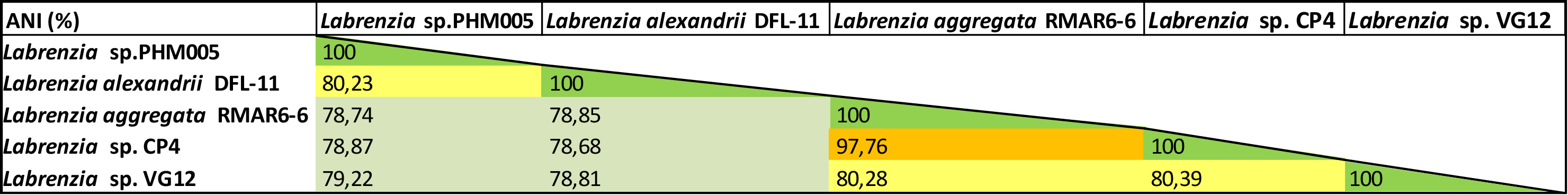

Supplement: FIGURE S3 — Average nucleotide identity (ANI) comparison shows interspecies and intraspecies relationships of five strains in the Labrenzia genera. Identity scale: Green = 100%; orange > 95%; yellow ≥ 80%; gray < 80%. [file Image_3.JPEG]

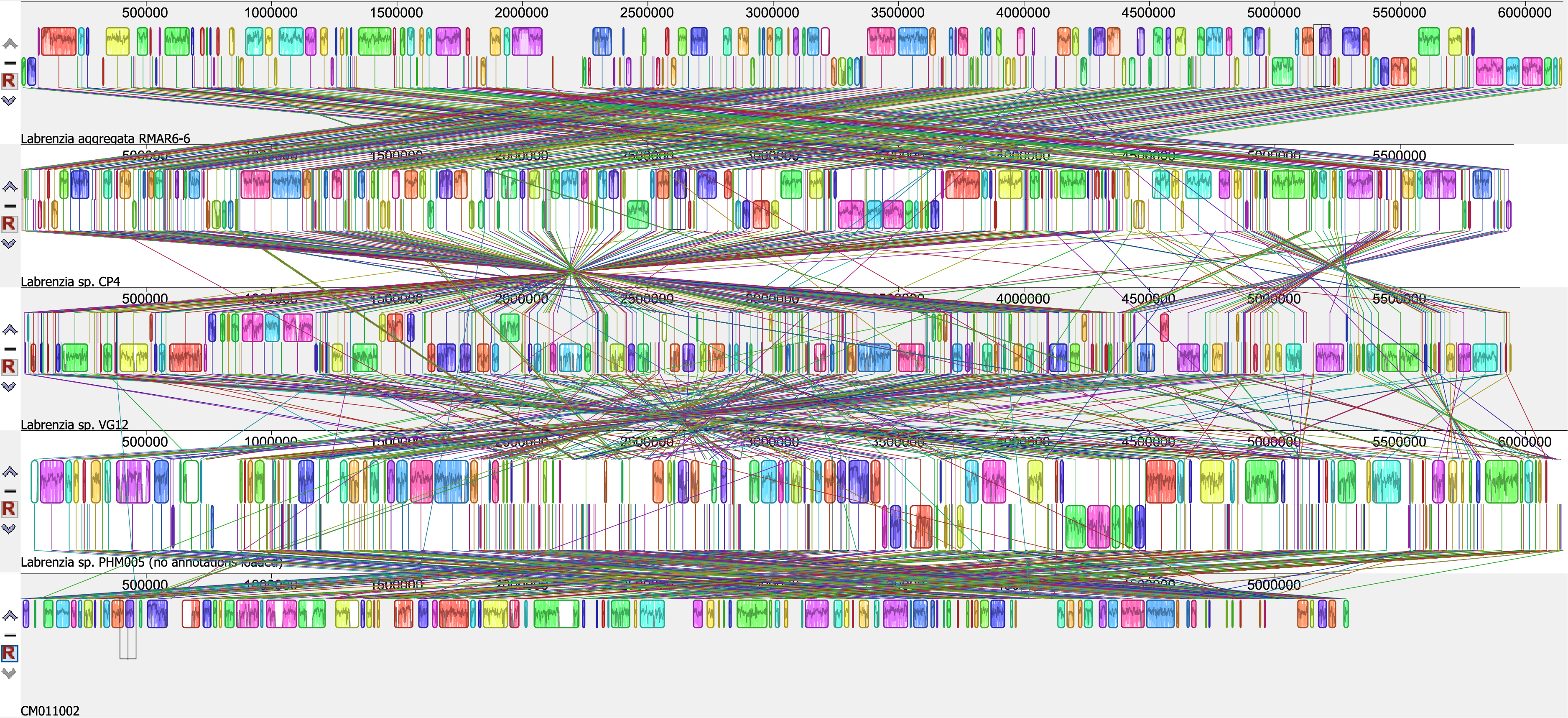

Supplement: FIGURE S4 — Whole genome alignment using by progressive Mauve algorithm (Genious version 10.0.2), from up to the bottom: L. aggregata RMAR-6, Labrenzia sp. CP4, Labrenzia sp. VG12, Labrenzia sp. PHM005, L. alexandrii DFL-11. [file Image_4.JPEG]

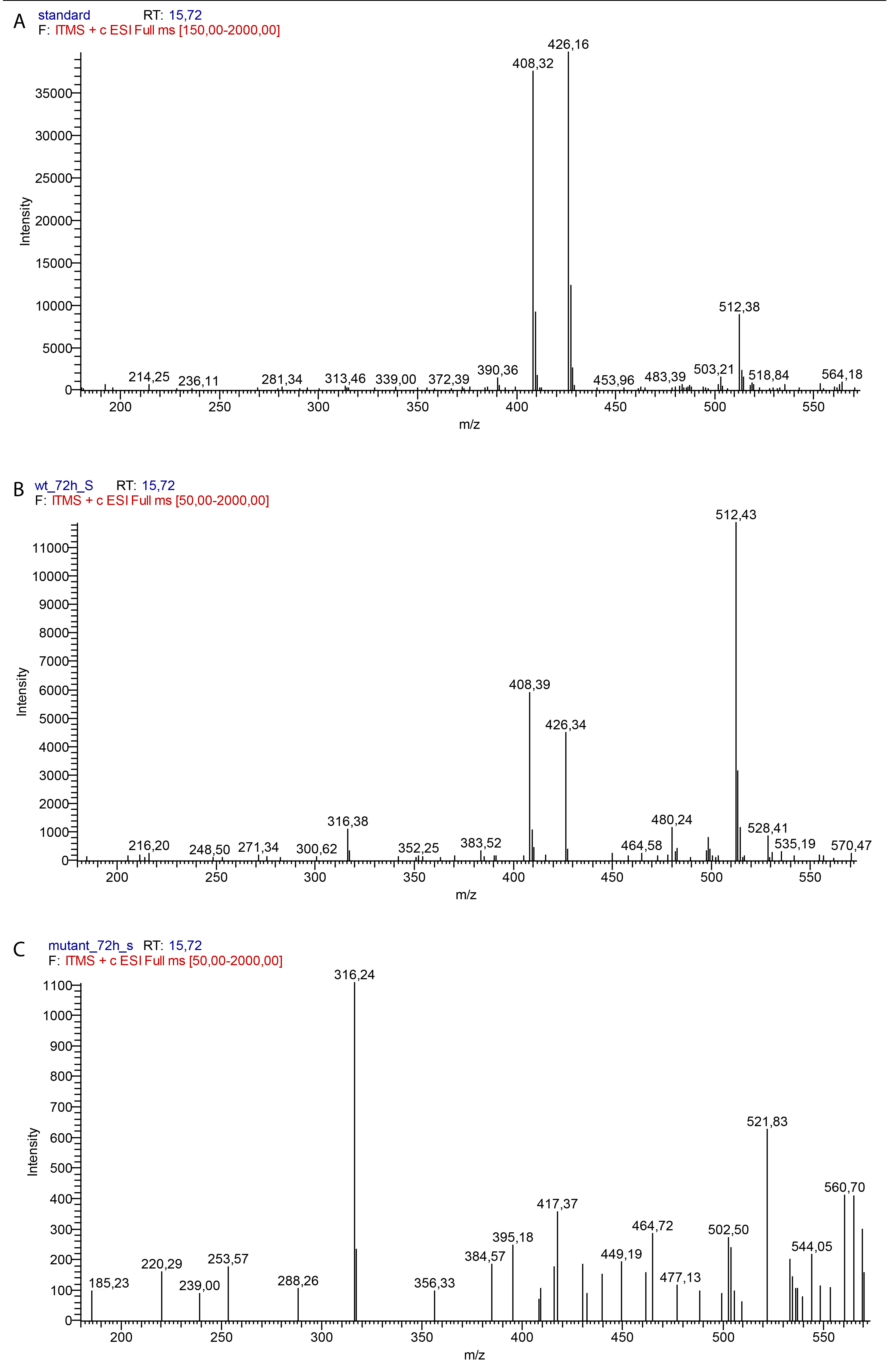

Supplement: FIGURE S5 — Specific MS ionization pattern of labrenzin in culture extracts of the wild type PHM005 and the PHM005ΔPKS4 mutant after 72 h of cultivation in MBM + vit medium. (A) Pure labrenzin (standard) fragmentation patern at RT = 15,72 min. (B) Labrenzin fragmentation patern at RT = 15,72 min extracted from the wild type strain culture. (C) Background fragmentation patern at RT = 15,72 min extracted from the PHM005ΔPKS4 mutant strain culture. [file Image_5.JPEG]
