# Supplementary material for: Genome of Labrenzia sp. PHM005 Reveals a Complete and Active Trans-AT PKS Gene Cluster for the Biosynthesis of Labrenzin
Source: Front Microbiol. 2019 Nov 7;10:2561. doi: 10.3389/fmicb.2019.02561 (PMC6855096; doi:10.3389/fmicb.2019.02561)
Supplement: TABLE S1 — Primers used for the cluster sequence correction. [file Table_1.DOCX]

**Table S1. Primers used for the cluster sequence correction**

| Primer | 5’- 3’ sequence |
| --- | --- |
| AF | CTTCCGGATACCGGCCAATC |
| AR | AAGGTGTCTGGGAGTCCAGT |
| BF | AATCTTGGGTCTGATCGCGG |
| BR | GCGCACATCGGAAAAGAACA |
| CF | AGAGTGATTTCCAACCGCCC |
| CR | ATTGGACGAGTCTGGGCAAC |
| DF | AATTTCATCCAGGGTGCCGT |
| DR | ATGCAGTAAACCCCGCCATT |
| EF | TTTCCGGGCGAAACTGTCTA |
| ER | GTGATCTGGCTTTGGAGGGT |
| FF | GCGGTTCCTGCAACAGAATG |
| FR | TATGCAGTGATCCGCCAGAC |

**Table S2. Primers used in qPCR**

| Primer | 5’- 3’ sequence |
| --- | --- |
| 4F | CGGAAGCGCCTGTAATCATG |
| 4R | GGGGTTCGGCTGACTATCAT |
| 5F | GACGGGTGGATCTTGGCTTT |
| 5R | TTATCAGCAACCAGACCGCC |
| 6F | CGATCTGGGCCGATATGCTT |
| 6R | CCTGGAGCGTTTTGAGAGGT |
| 7F | ATCAGATTAACCGGGCGTCC |
| 7R | GAGGCGATGCGAAATAAGGC |
| 8F | CAAAACTGTGCTGAGCCTGC |
| 8R | CGGGTGTTTGCCAAAGTGTC |
| 9F | GAGCACCATCAACATAGCGC |
| 9R | GGCTGAAACTCTGATTGCCG |
| 10F | TCTGAAATGACTGAGGCCGC |
| 10R | GGCATTGTATTCGCCCACAC |
| HKF | CACCACGACAAAAGAACCCG |
| HKR | GAGAACCTGGGCTTCGTTGA |

**Table S3. Coding sequences of p1BIR.**

| Putative protein | **BLAST homolog** | **Origin** | **Accession number** | **Cover/identity** | **Pfam domain** |
| --- | --- | --- | --- | --- | --- |
| Conjugal transfer coupling protein TraG | type IV secretory system conjugative DNA transfer family protein | *Geminicoccus roseus* | WP_084506409.1 | 50/39 | Type IV secretory system Conjugative DNA transfer |
| Site-specific recombinases, DNA invertase | recombinase family protein | *Acuticoccus kandeliae* | WP_108663905.1 | 99/76 | Resolvase, N terminal domain; helix-turn-helix domain |
| Abortive infection bacteriophage resistance protein | Abi family protein | *Sphingomonas melonis* | WP_020493507.1 | 88/58 | no hits |
| hypothetical protein | hypothetical protein | *Alteromonas* sp. W12 | WP_075176678.1 | 79/46 | no hits |
| hypothetical protein | hypothetical protein | *Sinorhizobium fredii* | WP_037456464.1 | 93/33 | no hits |
| hypothetical protein | hypothetical protein | *Bradyrhizobium ottawaense* | WP_091977009.1 | 86/43 | no hits |
| hypothetical protein | hypothetical protein X773_05775 | *Mesorhizobium* sp. LSJC285A00 | ESW87211.1 | 87/83 | no hits |
| Abortive infection bacteriophage resistance protein | Abi family protein | *Rhizobiales bacterium* | WP_113396613.1 | 92/54 | Abi-like protein |
| hypothetical protein | hypothetical protein A4X03_g9487 | *Tilletia caries* | OAI97131.1 | 90/43 | no hits |
| hypothetical protein | alpha/beta fold hydrolase | *Vibrio fluvialis* | WP_044362754.1 | 83/30 | no hits |
| hypothetical protein | hypothetical protein | *Labrenzia* sp. VG12 | WP_094072921.1 | 94/33 | no hits |
| RepA | plasmid replication initiator RepA | *Bacteroidetes bacterium* | PCJ62743.1 | 96/68 | Replication initiator protein A |
| ParA | chromosome partitioning protein ParA | *Tropicibacter naphthalenivorans* | WP_058249280.1 | 97/79 | VirC1 protein |
| hypothetical protein | hypothetical protein | *Acuticoccus yangtzensis* | WP_108676665.1 | 96/59 | no hits |
| Relaxase/Mobilisation nuclease domain-containing protein | Relaxase/Mobilisation nuclease domain-containing protein | *Mesorhizobium australicum* | SMH26052.1 | 86/35 | Relaxase/Mobilisation nuclease domain |
| MobC | plasmid mobilization relaxosome protein MobC | *Hyphomonas beringensis* | WP_034797206.1 | 54/49 | no hits |
| hypothetical protein | hypothetical protein TEF_21935 | *Rhizobiales bacterium* NRL2 | ANK83160.1 | 84/45 | no hits |

**Table S4. List of clusters homologous to those identified in *Labrenzia* sp.PHM005 by antiSMASH 4.0.**

| cluster type in PHM005 | genome location (bp) | Homologous gene cluster | MIBiG BGC-ID | % |
| --- | --- | --- | --- | --- |
| saccharide | 189368- 213810 | Kinamycin | BGC0000236_c1 | 5 |
| putative | 668100- 685508 | O-antigen | BGC0000782_c1 | 19 |
| saccharide | 686985- 711837 | Capsular polysaccharide | BGC0000758_c1 | 3 |
| [Transatpks](https://docs.antismash.secondarymetabolites.org/glossary/#transatpks)-Arylpolyene-[Nrps](https://docs.antismash.secondarymetabolites.org/glossary/#nrps) | 761226- 871081 | Oocydin A | BGC0001032_c1 | 21 |
| putative | 1010763- 1022201 | Succinoglycan | BGC0000802_c1 | 33 |
| putative | 1134775- 1143522 | Lipopolysaccharide | BGC0000774_c1 | 5 |
| putative | 1203168- 1209160 | Polyhydroxyalkanoate | BGC0000866_c1 | 50 |
| saccharide | 2871859- 2913267 | Capsular polysaccharide | BGC0000736_c1 | 16 |
| putative | 4250660- 4260523 | Xenocyloins | BGC0000189_c1 | 25 |
| putative | 5041493- 5048583 | Azinomycin_B | BGC0000960_c1 | 4 |
| putative | 5411153- 5416702 | Cepacian | BGC0000808_c1 | 18 |

**Table S5. Homologous gene clusters in other microorganisms according to ClusterBlast by antiSMASH 4.0.**

| cluster | genome location | Cluster Blast hits | NCBI sequence ID | % |
| --- | --- | --- | --- | --- |
| t1pks-saccharide | 480196 - 549637 | *Labrenzia alexandrii* DFL-11 | EQ973121 | 85 |
|  |  | *Rhizobium* sp. LC145 | LBHV01000004 | 25 |
| putative | 668100 - 685508 | *Pseudomonas* sp. GM74 PMI34 | AKJG01000048 | 17 |
|  |  | *Rhizobium tropici* CIAT 899 | CP004015 | 15 |
| saccharide | 686985 - 711837 | *Rhizobium etli bv. mimosae* str. IE4771 | CP006986 | 14 |
|  |  | *Azorhizobium caulinodans* ORS 571 | AP009384 | 11 |
| putative | 732631 - 741605 | *Kiloniella* sp. P1-1 | LANI01000017 | 27 |
|  |  | *Rhizobium rhizogenes* NBRC 13257 | BAYX01000006 | 22 |
| fatty_acid | 935479 - 956702 | *Rhodomicrobium vannielii* ATCC 17100 | CP002292 | 20 |
|  |  | *Rhizobium* sp. Leaf391 | LMQG01000042 | 17 |
| putative | 993628 - 1003997 | *Rhizobium* sp. Leaf311 | LMNZ01000009 | 12 |
|  |  | *Rhizobium rubi* NBRC 13261 | BBJU01000029 | 10 |
| putative | 2030747 - 2043034 | *Burkholderia* sp. Leaf177 | LMPF01000021 | 23 |
|  |  | *Bradyrhizobium valentinum* strain LmjM3 | LLXX01000143 | 20 |
| putative | 2140378 - 2156286 | *Bradyrhizobium elkanii* strain UASWS1015 | JXOF01000173 | 13 |
|  |  | *Bradyrhizobium* sp. YR681 PMI42 | AKIY01000259 | 10 |
| putative | 2225472 - 2235766 | *Mesorhizobium* sp. LNJC405B00 | AYWC01000002 | 15 |
|  |  | *Mesorhizobium* sp. Root554 | LMGA01000001 | 12 |
| putative | 2359048 - 2367711 | *Phaeobacter* sp. CECT 5382 | CYSG01000021 | 34 |
|  |  | *Bradyrhizobium* sp. DFCI-1 | AMFB01000012 | 21 |
| putative | 2379843 - 2396431 | *Rhizobium rubi* NBRC 13261 | BBJU01000023 | 17 |
| putative | 3516897 - 3522239 | *Neorhizobium galegae bv. officinalis* | CCRH01000015 | 17 |
| fatty_acid | 4522154 - 4543389 | *Sinorhizobium fredii* HH103 | HE616890 | 25 |
| putative | 4543774 - 4553183 | *Bradyrhizobium* sp. STM 3843 | CAFK01000193 | 16 |
| putative | 4658717 - 4682200 | *Mesorhizobium* sp. Root695 | LMHO01000015 | 20 |
| fatty_acid | 4699018 - 4719995 | *Phaeospirillum fulvum* MGU-K5 | AQPH01000012 | 48 |
|  |  | *Mesorhizobium* sp. Root102 | LMCP01000045 | 32 |
| putative | 4825217 - 4843781 | *Haematobacter missouriensis* strain CCUG 52307 | JFGS01000018 | 25 |
|  |  | *Bradyrhizobium* sp. LTSP849 | JYMR01000055 | 25 |

**Table S6. Domain specificity of the three PKS and mixed PKS/NRPS.**

| Domain | | Specificity |
| --- | --- | --- |
| PKS4 | GNAT |  |
|  | ACP | non-beta branching; active site serine |
|  | KS | active site cysteine and histidine |
|  | DH | no (HxxxGxxxxP) conserved motif |
|  | KR | stereochemistry: C2; conserved motif (GxGxxAxxxA) present |
|  | cMT | conserved motif (LExGxGxG) present |
|  | ACP | non-beta branching; active site serine |
|  | KS | active site cysteine and histidine |
|  | *trans*-AT docking |  |
|  | ECH |  |
|  | ACP | beta |
|  | ACP | beta |
|  | KS | active site cysteine and histidine |
|  | KR | conserved motif (GxGxxAxxxA) not present |
|  | ACP | non-beta branching; active site serine |
| PKS15 | KS | active site cysteine |
|  | DH | no (HxxxGxxxxP) conserved motif |
|  | ACP | non-beta branching; active site serine |
|  | C |  |
|  | A | pHMM: glycine; SANDPUMA: glycine |
|  | PCP |  |
|  | KS | active site cysteine and histidine |
|  | *trans*-AT docking |  |
|  | KR | conserved motif (GxGxxAxxxA) changed for (GxGxxGxxxA) |
|  | ACP | non-beta branching; active site serine |
|  | KS | active site cysteine and histidine |
|  | *trans*-AT docking |  |
|  | KR | stereochemistry: C2; conserved motif (GxGxxAxxxA) changed for (GxGxxGxxxT) |
|  | cMT | conserved motif (LExGxGxG) present |
|  | ACP | beta |
|  | KS | active site cysteine and histidine |
|  | *trans*-AT docking |  |
|  | DH | (HxxxGxxxxP) conserved motif present |
|  | PS |  |
|  | KR | conserved motif (GxGxxAxxxA) changed for (GxGxxGxxxA) |
|  | ACP | non-beta branching; active site serine |
|  | KS | active site cysteine and histidine |
|  | *trans*-AT docking |  |
|  | KR | conserved motif (GxGxxAxxxA) changed for (GxGxxGxxxA) |
|  | ACP | non-beta branching; active site serine |
|  | KS | active site cysteine and histidine |
|  | *trans*-AT docking |  |
| PKS13 | ACP | non-beta branching; active site serine |
|  | KS | active site cysteine and histidine |
|  | *trans*-AT docking |  |
|  | DH | (HxxxGxxxxP) conserved motif present |
|  | KR | stereochemistry: A1; conserved motif (GxGxxAxxxA) changed for (GxGxxGxxxA) |
|  | ACP | non-beta branching; active site serine |
|  | KS | active site cysteine and histidine |
|  | DH | no (HxxxGxxxxP) conserved motif |
|  | DH | (HxxxGxxxxP) conserved motif present |
|  | ACP | non-beta branching; active site serine |
|  | KS | active site cysteine and histidine |
|  | *trans*-AT docking |  |
|  | KR | conserved motif (GxGxxAxxxA) changed for (GxGxxGxxxA) |
|  | ACP | non-beta branching; active site serine |
|  | KS | active site cysteine |
|  | *trans*-AT docking |  |
|  | ACP | no conserved motif GxDS found |
|  | C |  |
|  | A | pHHM: arginine; SANDPUMA: alanine |
|  | PCP |  |
|  | TE | active site serine;conserved motifs (GxSxG) and (GxH) |

**Table S7. Four *Labrenzia* genomes aligned to *Labrenzia* sp. PHM005 using progressive Mauve algorithm individually.**

| *Labrenzia* sp. PHM005 aligned to: | minimum weight for Locally Collinear Blocks | |
| --- | --- | --- |
| *Labrenzia alexandrii* DFL-11 | 164 | |
| *Labrenzia aggregata* RMAR-6 | 42 | |
| *Labrenzia* sp. CP4 | 92 | |
| *Labrenzia* sp. VG12 | 72 |  |

**Table S8. BLAST homology of the *Labrenzia* sp. PHM005 *trans*- AT PKS gene cluster.**

| ORF | **Annotation (RAST)** | **BLAST homolog** | **Origin** | **accession number** | **cover/identity %** |
| --- | --- | --- | --- | --- | --- |
| HYP1 | DUF3089 domain-containing protein | DUF3089 domain-containing protein | *Labrenzia* sp. DG1229 | WP_051644561.1 | 100/65 |
| HYP2 | ParA family protein | ParA family protein | *Labrenzia marina* | WP_103225501.1 | 100/73 |
| HYP3 | DUF697 domain-containing protein | DUF697 domain-containing protein | *Labrenzia* sp. Alg231-36 | WP_108873020.1 | 100/81 |
| HYP4 | DUF697 domain-containing protein | DUF697 domain-containing protein | *Labrenzia* sp. Alg231-36 | WP_108873020.1 | 100/81 |
| HYP5 | prohibitin family protein | prohibitin family protein | *Stappia indica* | WP_067221789.1 | 98/62 |
| 1 | ABC transporter substrate-binding protein | ABC transporter substrate-binding protein | *Oceanibaculum indicum* | WP_008945422.1 | 91/37 |
| HYP6 | hypothetical protein | hypothetical protein | *Oceanibaculum indicum* | WP_008945421.1 | 95/62 |
| 2 | acyl carrier protein | acyl carrier protein PedN | Candidatus *Profftella armatura* | WP_020915408.1 | 95/44 |
| 3 | hydroxymethylglutaryl-CoA synthase family protein | 3-hydroxy-3-methylglutaryl-ACP synthase | *Clostridium beijerinckii* | WP_023973754.1 | 100/65 |
|  |  | PedP | symbiont bacterium of *Paederus fuscipes* | AAW33975.1 | 99/61 |
|  |  | 3-hydroxy-3-methylglutaryl-ACP synthase | *Methylocucumis oryzae* | WP_045779829.1 | 99/65 |
| 4 | trans- AT polyketide synthase type I | OnnB | symbiont bacterium of *Theonella swinhoei* | AAV97870.1 | 79/40 |
|  |  | putative type I polyketide synthase PedI | symbiont bacterium of *Paederus fuscipes* | AAR19304.1 | 96/42 |
| 5 | LLM class flavin-dependent oxidoreductase | luciferase | Methylocucumis oryzae | WP_045777999.1 | 100/64 |
|  |  | flavin-dependent oxygenase | *Nostoc* sp. *'Peltigera membranacea* cyanobiont' | ADA69238.1 | 100/60 |
|  |  | putative oxygenase PedJ | symbiont bacterium of *Paederus fuscipes* | AAR19305.1 | 100/61 |
|  |  | OnnC | symbiont bacterium of *Theonella swinhoei* | AAV97871.1 | 100/63 |
| 6 | methyltransferase domain-containing protein | OnnG | symbiont bacterium of *Theonella swinhoei* | AAV97875.1 | 99/51 |
|  |  | OnnD | symbiont bacterium of *Theonella swinhoei* | AAV97872.1 | 97/46 |
|  |  | hypothetical protein ETSY1_46125 (plasmid) | Candidatus *Entotheonella* sp. TSY1 | : ETX03779.1 | 99/51 |
|  |  | PedO | symbiont bacterium of *Paederus fuscipes* | AAW33974.1 | 97/43 |
|  |  | putative methyltransferase PedA | symbiont bacterium of *Paederus fuscipes* | AAS47557.1 | 97/47 |
|  |  | SAM-dependent methyltransferase, partial | *Methylocucumis oryzae* | WP_052700174.1 | 72/58 |
| 7 | cytochrome P450 | cytochrome P450 | *Labrenzia alba* | WP_055112014.1 | 97/54 |
|  |  | cytochrome P450 | *Oceanibaculum indicum* | WP_008944496.1 | 98/54 |
|  |  | cytochrome P450 monooxygenase | *Magnetospirillum gryphiswaldense* MSR-1 | CAM75136.1 | 98/48 |
| 8 | FMN-dependent oxidoreductase | FMN-dependent oxidoreductase PedB | symbiont bacterium of *Paederus fuscipes* | AAS47558.1 | 98/56 |
|  |  | putative FMN-dependent oxidoreductase, PedB-like protein | Candidatus *Profftella armatura* | WP_020915402.1 | 98/52 |
| 9 | acyltransferase domain-containing protein | acyltransferase domain-containing protein | Methylobacter tundripaludum | WP_104425066.1 | 98/41 |
|  |  | polyketide biosynthesis acyltransferase | Candidatus *Profftella armatura* | WP_020915461.1 | 92/34 |
|  |  | putative acyltransferase PedC | symbiont bacterium of *Paederus fuscipes* | AAS47559.1 | 98/35 |
| 10 | ACP S-malonyltransferase | malonyl CoA-acyl carrier protein transacylase | *Burkholderia gladioli* | WP_036053944.1 | 98/51 |
|  |  | malonyl CoA-acyl carrier protein transacylase | *Methylocucumis oryzae* | WP_045778127.1 | 98/50 |
|  |  | malonyl CoA-acyl carrier protein transacylase | Candidatus *Entotheonella* sp. (ex. *Theonella swinhoei*) | AKQ22695.1 | 98/52 |
|  |  | putative acyltransferase PedD | symbiont bacterium of *Paederus fuscipes* | AAS47563.1 | 95/51 |
| 11 | enoyl-CoA hydratase/isomerase | enoyl-CoA hydratase | *Oceanibaculum indicum* | WP_008944492.1 | 94/50 |
|  |  | enoyl-CoA hydratase | *Labrenzia* sp. DG1229 | WP_029061353.1 | 97/43 |
|  |  | PedL | symbiont bacterium of *Paederus fuscipes* | AAW33971.1 | 91/43 |
| 12 | polyketide beta-ketoacyl:ACP synthase | polyketide beta-ketoacyl:ACP synthase | *Oceanibaculum indicum* | WP_008944493.1 | 98/44 |
|  |  |  |  |  |  |
|  |  | Polyketide biosynthesis malonyl-ACP decarboxylase PksF | *Labrenzia alba* | CTQ66097.1 | 97/42 |
|  |  | PedM* | symbiont bacterium of *Paederus fuscipes* | AAW33972.1 | 100/27 |
| 13 | mixed type I polyketide synthase - peptide synthetase | mixed type I polyketide synthase - peptide synthetase | symbiont bacterium of *Paederus fuscipes* | AAS47562.1 | 99/42 |
|  |  | non-ribosomal peptide synthetase | *Magnetovibrio blakemorei* | WP_069956861.1 | 98/36 |
|  |  | Polyketide synthase PksN | *Labrenzia alba* | CTQ74038.1 | 98/35 |
| 14 | monooxygenase | putative FAD-dependent monooxygenase | symbiont bacterium of *Paederus fuscipes* | AAS47561.1 | 94/73 |
|  |  | flavin-containing monooxygenase PedG-like protein | Candidatus *Profftella armatura* | WP_020915400.1 | 98/71 |
|  |  | monooxygenase | *Labrenzia alba* | WP_055112032.1 | 97/62 |
| 15 | mixed type I polyketide synthase - peptide synthetase | mixed type I polyketide synthase/nonribosomal peptide synthetase | symbiont bacterium of *Paederus fuscipes* | AAS47564.1 | 99/42 |
|  |  | non-ribosomal peptide synthetase | *Methylobacter tundripaludum* | WP_104425077.1 | 96/46 |
|  |  | OnnI, partial | symbiont bacterium of *Theonella swinhoei* | AAV97877.1 | 92/46 |
| 16 | methyltransferase domain-containing protein | OnnH | symbiont bacterium of *Theonella swinhoei* | AAV97876.1 | 99/43 |
|  |  | putative methyltransferase PedE | symbiont bacterium of *Paederus fuscipes* | AAS47560.1 | 98/51 |
|  |  | class I SAM-dependent methyltransferase | *Methylocucumis oryzae* | WP_045777863.1 | 94/57 |
| 17 | ATP-binding cassette domain-containing protein | hypothetical protein ETSY1_10275 | Candidatus *Entotheonella* sp. TSY1 | ETX00685.1 | 99/33 |
|  |  | cyclic peptide transporter | *Oceanibaculum indicum* | WP_008944488.1 | 98/52 |
|  |  | fused multidrug transport subunits of ABC superfamily transporter: permease component/ATP-binding protein | *Magnetospirillum gryphiswaldense* | WP_024079861.1 | 99/45 |
| 18 | cyclic peptide export ABC transporter | cyclic peptide transporter | *Oceanibaculum indicum* | WP_008944489.1 | 99/51 |
|  |  | fused multidrug transport subunits of ABC superfamily transporter: permease component/ATP-binding protein | *Magnetospirillum gryphiswaldense* | WP_024079861.1 | 95/40 |
|  |  | ABC transporter, ATP-binding/permease protein | *Magnetospirillum gryphiswaldense* MSR-1 | CAM74928.1 | 98/51 |
| 19 | 4'-phosphopantetheinyl transferase superfamily protein | 4'-phosphopantetheinyl transferase superfamily protein | *Methylobacter tundripaludum* | WP_104424065.1 | 97/42 |
|  |  | 4'-phosphopantetheinyl transferase | Candidatus *Profftella armatura* | WP_020915412.1 | 82/31 |
|  | * BLAST Global Alignment |  |  |  |  |
